# Regulatory Mechanisms and Functional Roles of Hypoxia-Induced Long Non-Coding RNA *MTORT1* in Breast Cancer Cells

**DOI:** 10.3389/fonc.2021.663114

**Published:** 2021-06-01

**Authors:** Yi-Chun Cheng, Li-Yu Su, Li-Han Chen, Tzu-Pin Lu, Eric Y. Chuang, Mong-Hsun Tsai, Li-Ling Chuang, Liang-Chuan Lai

**Affiliations:** ^1^ Institute of Physiology, College of Medicine, National Taiwan University, Taipei, Taiwan; ^2^ Institute of Fisheries Science, College of Life Science, National Taiwan University, Taipei, Taiwan; ^3^ Institute of Epidemiology and Preventive Medicine, National Taiwan University, Taipei, Taiwan; ^4^ Bioinformatics and Biostatistics Core, Center of Genomic and Precision Medicine, National Taiwan University, Taipei, Taiwan; ^5^ Graduate Institute of Biomedical Electronics and Bioinformatics, National Taiwan University, Taipei, Taiwan; ^6^ Collage of Biomedical Engineering, China Medical University, Taichung, Taiwan; ^7^ Institute of Biotechnology, National Taiwan University, Taipei, Taiwan; ^8^ School of Physical Therapy and Graduate Institute of Rehabilitation Science, College of Medicine, Chang Gung University, Taoyuan, Taiwan; ^9^ Department of Physical Medicine and Rehabilitation, Chang Gung Memorial Hospital, Taoyuan, Taiwan

**Keywords:** long noncoding RNA, mitochondria, hypoxia, function, microRNA, breast cancer

## Abstract

Long non-coding RNAs (lncRNAs) have been found to participate in multiple genetic pathways in cancer. Also, mitochondria-associated lncRNAs have been discovered to modulate mitochondrial function and metabolism. Previously, we identified oxygen-responsive lncRNAs in MCF-7 breast cancer cells under different oxygen concentrations. Among them, a novel mitochondria-encoded lncRNA, *mitochondrial oxygen-responsive transcript 1* (*MTORT1*), was chosen for further investigation. Nuclear, cytoplasmic, and mitochondrial fractionation assays were performed to evaluate the endogenous expression levels of *MTORT1* in breast cancer cells. *In vitro* proliferation and migration assays were conducted to investigate the functions of *MTORT1* in breast cancer cells by knockdown of *MTORT1*. RNA immunoprecipitation and luciferase reporter assays were used to examine the physical binding between *MTORT1* and microRNAs. Our results showed that *MTORT1* had low endogenous expression levels in breast cancer cells and was mainly located in the mitochondria. Knockdown of *MTORT1* enhanced cell proliferation and migration, implying a tumor suppressor role of this novel mitochondrial lncRNA. *MTORT1* served as sponge of *miR-26a-5p* to up-regulate its target genes, *CREB1* and *STK4*. Our findings shed some light on the characterization, function, and regulatory mechanism of the novel hypoxia-induced mitochondrial lncRNA *MTORT1*, which functions as a microRNA sponge and may inhibit breast cancer progression. These data suggest that *MTORT1* may be a candidate for therapeutic targeting of breast cancer progression.

## Introduction

Solid tumors often acquire a state of limited oxygen and nutrients during tumor progression because of rapid tumor growth and poor vascular distribution. To adapt to the decreased oxygen availability, a series of genomic pathways are activated, mainly by hypoxia inducible factors (HIFs) (1). Signaling pathways of the genes induced by HIF-1 include epithelial-mesenchymal transition, apoptosis, angiogenesis, glycolysis, and others (2–4). The activation of these signaling pathways allows tumor cells to accommodate their hypoxic microenvironment, promoting malignant progression, and is partially responsible for their resistance to chemotherapy and radiation therapy (5, 6). Although hypoxia is an essential factor in cancer progression, its regulatory mechanisms are still not clear.

Cancer cells have been observed to undergo an increase in glycolysis and lactate production in the presence of high oxygen (7, 8). The impairment of aerobic respiration and a reliance on glycolytic metabolism in cancer cells might help cancer cells live in low oxygen conditions, like hypoxia. The model of aerobic glycolysis incorporates the biosynthesis of molecules and organelles to replicate new cells, because the intermediates of the glycolytic pathway are used in various biosynthetic pathways, such as nucleoside and amino acid generation (9). However, cancer cells still maintain a dynamic equilibrium between glycolysis and oxidative phosphorylation (10, 11). The cooperation of glycolysis and oxidative phosphorylation gives cancer cells more metabolic flexibility to survive in changing environments, like hypoxia, and can cause chemoresistance during chemotherapy (12). In addition, mitochondria are involved in other adaptive mechanisms, such as apoptosis, homeostasis, and innate immunity (12, 13). Although mitochondria play a central role in multiple functions in tumor progression, the mitochondria-related mechanisms remain unclear.

Non-coding RNAs, which have limited potential to translate, have been proposed in recent years to cause diseases such as cancer, due to their dysregulated expression (14). Among the non-coding RNAs, the RNA transcripts with lengths greater than 200 nucleotides are classified as long non-coding RNAs (lncRNAs). Expression of lncRNA has been found to be tissue-specific and is implicated in a variety of pathways, including tumorigenesis, cell cycle control, apoptosis, and migration (15, 16). Numerous studies have revealed different functions of lncRNA. For instance, lncRNAs act as microRNA (miRNA) sponges by interacting with the miRNAs to regulate post-transcriptional degradation of their mRNA targets. LncRNAs can also bind to transcription factors and prevent or assist with their location on the promoter of their target DNA. LncRNAs also modulate pre-mRNA splicing (17, 18).

In mitochondria, lncRNAs can be either mitochondria-encoded (mtlncRNAs) or nucleus-encoded (19). Three mtlncRNAs, *lncND5*, *lncND6*, and *lncCyt b*, are complementary to the mitochondrial genes *ND5*, *ND6*, and *Cyt b*, respectively (20). The function of these mtlncRNAs is to form duplexes with their respective complementary mRNAs to stabilize them or regulate their expression (21). In addition, the sense noncoding mitochondrial RNAs (SncmtRNAs) and anti-sense noncoding mitochondrial RNAs (ASncmtRNAs) are located in both the mitochondria and the nucleus, and are known as retrograde signals communicating between mitochondria and nucleus (21, 22). The discovery of mtlncRNAs suggested the complexity of molecular regulation in cells. However, the effects of mtlncRNAs in tumors are not fully understood.

Previously, our lab identified a set of oxygen-responsive lncRNAs in MCF-7 breast cancer cells (23), but many of their functions were unknown. The purpose of this study was to investigate the functional role and regulatory mechanism of a novel mtlncRNA, *mitochondrial oxygen-responsive transcript 1* (*MTORT1*). We found that *MTORT1* could inhibit the proliferation and migration of breast cancer cells, and could serve as a miRNA sponge to up-regulate the nucleus-encoded genes, *CREB1* and *STK4*, by binding to *miR-26a-5p.*

## Materials and Methods

### Cell Culture and Treatments

Breast cancer cells (MCF-7, ZR-75-30, MDA-MB-231) and embryonic cells (HEK293T) were cultured in Dulbecco’s Modified Eagle Medium (DMEM) (GIBCO, Carlsbad, CA, USA) with 10% fetal bovine serum (FBS) (HyClone, Logan, UT, USA) and 1% penicillin-streptomycin-amphotericin solution (PS) (GIBCO). Human mammary epithelial cells (MCF-10A) were cultured in DMEM/Nutrient Mixture F-12 (GIBCO) with 5% horse serum, 20 ng/ml epidermal growth factor, 0.5 mg/ml hydrocortisone (Sigma, Saint Louis, MO, USA), 100 ng/ml cholera toxin (Sigma), 10 μg/ml insulin (Sigma), and 1% PS. The cells were incubated at 37°C in a humidified incubator with 5% CO_2_. To identify oxygen-responsive lncRNA, MCF-7 and MDA-MB-231 cells were cultured in a hypoxic chamber (InVivO2-200, Ruskinn Technology, Bridgend, UK) filled with 0.5% O_2_, 5% CO_2_ and 94.5% N_2_ for 24 h. After incubation under hypoxia, cells were moved to a humidified incubator with 21% O_2_ and 5% CO_2_ for 24 h to mimic re-oxygenation. In addition, MCF-7 cells were treated with 200 μM cobalt (II) chloride (CoCl_2_) (Sigma) to mimic hypoxic conditions.

### Cell Line Authentication

Cell experiments were performed on cells that were passaged less than 20 times and were routinely tested for mycoplasma using PCR Mycoplasma Detection Kit (ABM Inc., Vancouver, Canada). The cell lines were purchased from and authenticated by the Bioresource Collection and Research Center, Food Industry Research and Development Institute (Hsinchu, Taiwan).

### RNA Interference

To knock down *MTORT1* expression, cells were transfected with three small interfering RNAs (siRNAs) (CCAUGAAUAUUGUACGGUATT, GCAAUCAACCCUCAACUAUTT, CCACCAUCCUCCGUGAAAUTT) (BIOTOOLS, New Taipei City, Taiwan) or the control siRNA (UUCUCCGAACGUGUCACGUTT) using Lipofectamine RNAiMAX (Invitrogen, Carlsbad, CA, USA) according to the manufacturer’s protocol. MCF-7 cells were seeded in 12-well plates at a density of 7 × 10^4^ cells/well and transfected with siRNA for 48 h; MDA-MB-231 were seeded 1.5 × 10^5^ cells in 6-wells and transfected with siRNA for 24 h. *MTORT1* expression were checked by quantitative Reverse-Transcription-PCR (RT-PCR).

### RNA Extraction, Reverse Transcription and Quantitative RT-PCR

Total RNA was extracted using NucleoZOL reagent (Machery-Nagel, Düren, Germany) according to the manufacturer’s protocol, and reverse-transcribed to complementary DNA (cDNA) by High-Capacity cDNA Reverse Transcription Kit (Applied Biosystems, Carlsbad, CA, USA). MiRNA was reverse-transcribed using SuperScript IV Reverse Transcriptase (Invitrogen) with the primers from **Table 1**. Complementary DNA acted as a template to measure gene expression by quantitative PCR with OmicsGreen qPCR MasterMix (OmicsBio, New Taipei City, Taiwan) with the primers in **Table 1**, performed on a StepOnePlus Real-Time PCR System (Thermo Fisher). Each reaction was done in triplicate, and the relative gene expression was normalized to 18S rRNA or U6 using the 2^-ΔΔCt^ method.

**Table 1:** The primers for reverse transcription and quantitative RT-PCR.

Gene/miRNA	Primer	Sequence (5’ to 3’)
**Reverse Transcription**		
*miR-26a-5p*	GTTGGCTCTGGTGCAGGGTCCGAGGTATTCGCACCAGAGCCAACAGCCTATC	
*miR-26b-5p*	GTTGGCTCTGGTGCAGGGTCCGAGGTATTCGCACCAGAGCCAACACCTATCC	
*miR-338-5p*	GTTGGCTCTGGTGCAGGGTCCGAGGTATTCGCACCAGAGCCAACCACTCA	
*miR-1293*	GTTGGCTCTGGTGCAGGGTCCGAGGTATTCGCACCAGAGCCAACGCACAAAT	
*miR-1297*	GTTGGCTCTGGTGCAGGGTCCGAGGTATTCGCACCAGAGCCAACCACCTG	
*miR-181c*	GTTGGCTCTGGTGCAGGGTCCGAGGTATTCGCACCAGAGCCAACACTCAC	
*U6* snRNA	CGCTTCACGAATTTGCGTGTCAT	
**Quantitative RT-PCR**		
*MTORT1*	Forward	CTCACCCATCAACAACCGCT
	Reverse	GTGGCTTTGGAGTTGCAGTT
*HIF1A*	Forward	GCAGCAACGACACAGAAACT
	Reverse	TGGGTGAGGGGAGCATTACA
*HIF2A*	Forward	TCCATCATGCGACTGGCAAT
	Reverse	GTCACCACGGCAATGAAACC
*PIK3R2*	Forward	AGGCCATTGAAAGGACAGGG
	Reverse	GTGCCAGCAGGAAGCTCTTA
*FNBP1*	Forward	GGCTTTCTCTCAAGCTGGGT
	Reverse	TGTGATCCAAACTGGCTGGG
*CSK*	Forward	GGTCAGCGACTTTGGTCTCA
	Reverse	TCCGAAACTCCACACGTCAG
*PPP1R14B*	Forward	GCCTCAACCTAGAGGAGTGGAT
	Reverse	GCATCGTCACTCTCCATGTCCA
*PPP1R3C*	Forward	GCGTTGTGTTTGCTGACTCC
	Reverse	CGGTTGAAGGCTGAGGGAAAT
*RAC1*	Forward	CGGTGAATCTGGGCTTATGGGA
	Reverse	GGAGGTTATATCCTTACCGTACG
*GAPDH*	Forward	AACGGGAAGCTTGTCATCAATGGAAA
	Reverse	GCATCAGCAGAGGGGGCAGAG
*U6* snRNA	Forward	GCTTCGGCAGCACATATACTAAAAT
	Reverse	CGCTTCACGAATTTGCGTGTCAT
16S rRNA	Forward	GATGGTGCAGCCGCTATTAAA
	Reverse	CTTGGGTGGGTGTGGGTATAA
18S rRNA	Forward	TCAACTTTCGATGGTAGTCGCCGT
	Reverse	TCCTTGGATGTGGTAGCCGTTTCT
*miR-26a-5p*	Forward	GCGGCGGTTCAAGTAATCCAG
*miR-26b-5p*	Forward	GCGGCGGTTCAAGTAATTCA
*miR-338-5p*	Forward	AACAATATCCTGGTGC
*miR-1293*	Forward	GCGGTGGGTGGTCUGGA
*miR-1297*	Forward	GCGGCGGTTCAAGTAATTCA
*miR-181c*	Forward	GGGAACATTCAACCTGTCG
miRNA universal	Reverse	GTGCAGGGTCCGAGGT

### Isolation of Mitochondrial, Cytoplasmic, and Nuclear Fractions

To determine the subcellular distribution of *MTORT1*, mitochondrial, cytoplasmic, and nuclear fractions were separated using a Cell Fractionation Kit (Abcam, Cambridge, England) according to the manufacturer’s instructions. In brief, 3.3 × 10^6^ cells were harvested by centrifugation at 300 × g for 3 min and resuspended in 1 ml 1× buffer A containing 0.0625% detergent I. After incubation for 10 min on a rotator at room temperature, samples were centrifuged at 5,000 × g for 1 min at 4°C. The supernatant was the cytosolic fraction. The pellets were resuspended in 1 ml 1× buffer A containing 2% detergent II and incubated for 15 min on a rotator at room temperature. Samples were centrifuged again at 5,000 × g for 1 min at 4°C. The supernatant was the mitochondrial fraction, and the pellet was the nuclear fraction. The RNA of these fractions was extracted using a HiYield™ Total RNA extraction kit (ArrowTec) according to the manufacturer’s instructions, reverse-transcribed, and measured by quantitative RT-PCR. The protein of these fractions was measured by western blotting.

### Microarray Analysis

Total RNA from cells transfected with 100 nM siRNA against *MTORT1* was amplified by an Illumina™ TotalPrep™ RNA Amplification Kit (Ambion) to revere-transcribe it to cDNA with a T7 promoter. When converting the single-stranded cDNA into a double-stranded cDNA, the second strand of cDNA was synthesized using DNA polymerase while the RNA was simultaneously degraded by RNase H. *In vitro* transcription was then conducted using the double-stranded cDNA as a template and T7 RNA polymerase to synthesize multiple copies of biotinylated complementary RNA (cRNA). After the cRNA was hybridized to Illumina Human HT-12 v4 BeadChips (Illumina) at 58°C for 16 h, the BeadChip was washed and stained with streptavidin-Cy3 dye. The intensity of the fluorescence across the surface of the chip was detected by the Illumina BeadArray Reader, and the results were analyzed using BeadStudio v3.1 software. After scanning, the intensity data from the BeadChips were analyzed using the commercial software Partek (Partek, St. Charles, MO, USA). The probe intensities were normalized by a quantile algorithm based on the intensity distribution across all chips. The gene expression profiles were evaluated by principal component analysis. The visual representation of expression profiles was generated by hierarchical clustering analysis using the Genesis 1.7.7 program (Graz University of Technology, Graz, Austria). Gene-gene interaction networks, biological functions, and pathways of these differentially expressed genes were analyzed by Ingenuity Pathway Analysis (Ingenuity Systems Inc., Redwood City, CA, USA). Microarray data from this study have been submitted to the GEO (Gene Expression Omnibus) database (accession number GSE157060).

### RNA Immunoprecipitation (RIP)

To determine the interaction between *MTORT1* and Argonaute 2 (AGO2), the Magna RIP Kit (Millipore) was used according to the manufacturer’s instructions. A total of 2 × 10^7^ cells was harvested with 0.05% trypsin-EDTA (GIBCO) and lysed in 100 μl RIP lysis buffer containing a proteinase inhibitor cocktail and RNase inhibitor (Millipore). The lysate was centrifuged at 13,000 × g for 10 min. Then, the supernatant was added to 900 μl RIP immunoprecipitation buffer with 2 μg anti-AGO2 antibodies (Boster Biological Technology, Pleasanton, CA, USA) that were pre-bound on magnetic beads for overnight agitation at 4°C. Ten percent of the supernatant was saved as input. Beads were washed 6 times with RIP wash buffer and treated with proteinase K at 55 °C for 30 min. RNA was extracted using NucleoZOL reagent (Machery-Nagel) and reverse-transcribed, and the relative gene expression level was measured by quantitative RT-PCR.

### Luciferase Reporter Assay

To determine the binding effect of *miR-26a-5p* on *MTORT1*, HEK293T cells were seeded in 24-well plates at a density of 4 × 10^4^ cells/well and co-transfected with 50 ng pMIR-REPORT-*MTORT1* or pMIR-REPORT-*MTORT1* mutant, 0.025 nmol *miR-26a-5p* mimics (GE Healthcare Dharmacon, Lafayette, CO, USA), and 1 ng *Renilla* luciferase plasmid (pGL4.74 [hRluc/TK]) using jetPRIME (Polyplus-transfection, Illkirch, France). In addition, miRNA mimic *cel-miR-67* (5’ - UCACAACCUCCUAGAAAGAGUAGA – 3’) (GE Healthcare Dharmacon) was used as the negative control. After 48 h, cells were lysed in 100 μl cell lysis buffer (92.8 mM K_2_HPO_4_, 9.2 mM KH_2_PO_4_ and 0.2% Triton X-100 in ddH_2_O), and the luciferase activity was measured using the Dual-Glo luciferase reporter assay system (Promega, Fitchburg, WI, USA).

### MTT Assay

MCF-7 and MDA-MB-231 cells were seeded in 6-well plates at a density of 1.5 × 10^5^ cells/well. MCF-7 cells were transfected with siRNA for 48 h and MDA-MB-231 cells for 24 h. Then, MCF-7 and MDA-MB-231 cells were passaged and seeded in 96-well plates at a density of 5,000 cells/well. One hundred µl 3-(4,5-dimethylthiazol-2-yl)-2,5-diphenyltetrazolium bromide (MTT) (EMD Biosciences, San Diego, CA, USA) were added to each well and incubated for 1 h at 6, 24, 48, 72, and 96 hours after seeding. The absorbance was then measured using an enzyme-linked immunosorbent assay (ELISA) reader (Thermo Scientific) at 570 nm. The cell proliferation rate was normalized to the absorbance measured at 6 h.

### Wound Healing Assay

MCF-7 and MDA-MB-231 cells were seeded in 6-well plates at a density of 1.5 × 10^5^ cells/well. MCF-7 cells were transfected with siRNA for 48 h and MDA-MB-231 cells for 24 h. Then, MCF-7 cells were passaged and seeded in the well of an Ibidi Culture-Insert (Ibidi, Martinsried, Germany) at a density of 2.5 × 10^4^ cells/well, and MDA-MB-231 cells were seeded at a density of 4 × 10^4^ cells/well. After cells were incubated overnight, the culture-inserts were removed gently with sterile tweezers to create a cell-free gap. The gap area was imaged by microscope at 0, 12, and 24 h and quantified using ImageJ 1.51 software (24).

### Colony Formation Assay

MCF-7 and MDA-MB-231 cells were seeded in 6-well plates at a density of 1.5 × 10^5^ cells/well. MCF-7 cells were transfected with siRNA for 48 h and MDA-MB-231 cells for 24 h. Then, MCF-7 and MDA-MB-231 cells were passaged and seeded in 6-well plates at a density of 500 cells/well. After incubation for 2 weeks, cells were fixed with 800 μl fixing solution containing 75% methanol and 25% acetate (Sigma) and stained with 0.1% crystal violet (Sigma). Colonies with cell numbers greater than 50 were calculated and quantified using ImageJ 1.51 software.

### Statistical Analysis

All results are presented as the means ± SDs from at least 3 independent experiments. All data were analyzed by Student’s t test to assess the differences between each group.

## Results

Previously, our lab used next generation sequencing (NGS) technology to identify differentially expressed lncRNAs in breast cancer MCF-7 cells under normoxic, hypoxic, and re-oxygenated conditions (23). The expression data can be downloaded from Gene Expression Omnibus (GEO, GSE84167). Among the hypoxia responsive lncRNAs, one RNA transcript, NONHSAT135851.2 (http://www.noncode.org/show_rna.php?id=NONHSAT135851&version=2&utd=1#) according to the nomenclature in the NONCODE database (25), was significantly up-regulated under hypoxia and down-regulated under re-oxygenation. Because NONHSAT135851.2 was located in the human mitochondrial genome and had different expression levels under different oxygen concentrations, we renamed it *mitochondrial oxygen responsive transcript 1* (*MTORT1*), which it is called hereafter.

**Figure 1:**
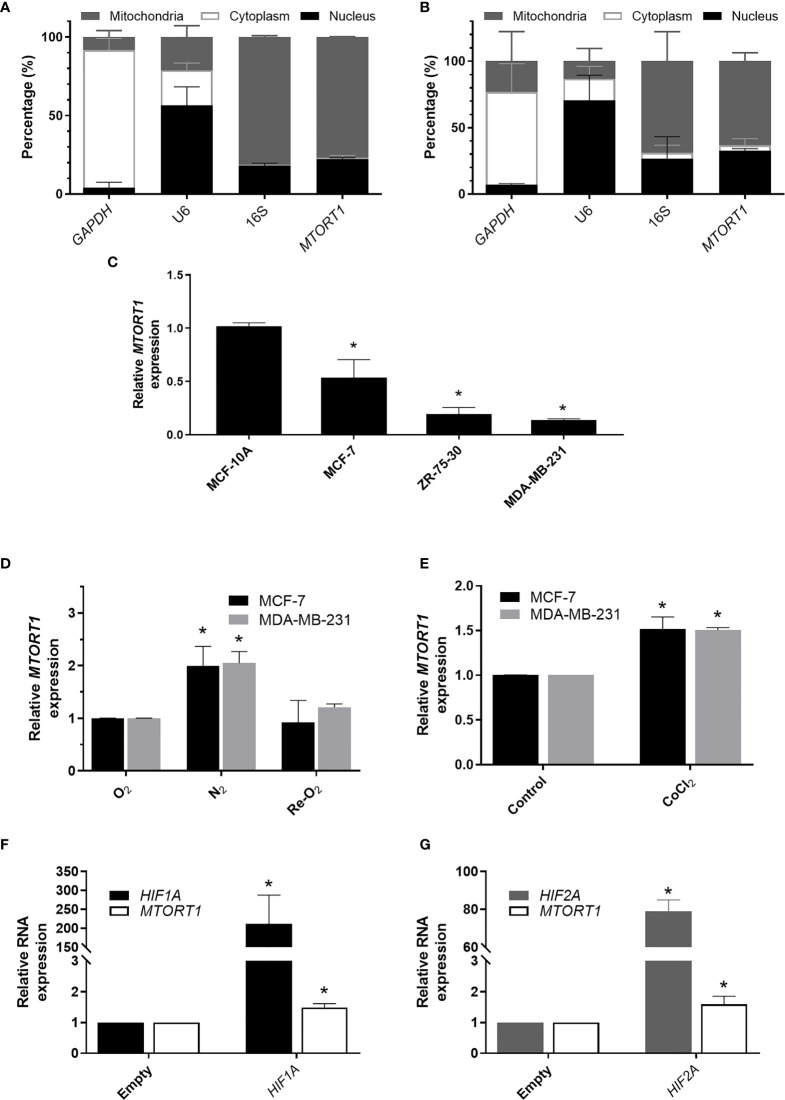
The mitochondrial lncRNA *MTORT1* is up-regulated under hypoxia and down-regulated under re-oxygenation in breast cancer cells. **(A, B)** The subcellular distribution of *MTORT1* in MCF-7 cells under normoxia **(A)** or hypoxia **(B)**. Relative abundance of RNA was measured by quantitative RT-PCR and normalized to total RNA. *GAPDH*, a cytosolic marker; *U6*, a nuclear marker; *16S*, a mitochondrial marker. **(C)** Relative endogenous expression levels of *MTORT1* in breast cancer cells, including MCF-7, ZR-75-30, and MDA-MB-231 cells, and MCF-10A breast epithelial cells. The expression levels were measured by quantitative RT-PCR and normalized to 18S rRNA. The relative expression levels in each cell line were compared with MCF-10A. **(D)** Relative expression levels of *MTORT1* in MCF-7 and MDA-MB-231 cells under different oxygen concentrations were measured by quantitative RT-PCR and normalized to 18S rRNA. The relative expression levels of each condition were compared with the normoxic group. **(E)** Relative expression levels of *MTORT1* in MCF-7 and MDA-MB-231 cells treated with 200 μM CoCl_2_ for 24 h. **(F)** Relative expression levels of *HIF-1A* and *MTORT1* in MCF-7 cells overexpressing HIF-1α P402A/P564A mutant, which is resistant to VHL-mediated ubiquitination and degradation. **(G)** Relative expression of *HIF-2A* and *MTORT1* in MCF-7 cells overexpressing HIF-2α P405A/P531A mutant, which is resistant to VHL-mediated ubiquitination and degradation. The results are means ± SDs (n = 3). *P < 0.05.

First, we separated the mitochondrial, cytoplasmic, and nuclear fractions to determine the subcellular distribution of *MTORT1* under normoxia (**Figure 1A**) or hypoxia (**Figure 1B**) in MCF-7 cells. As expected, the data showed that *MTORT1* is mainly located in mitochondria. Next, we determined the endogenous expression of *MTORT1* in breast cancer cells, including MCF-7 (luminal A), ZR-75-30 (luminal B) and MDA-MB-231 (triple negative) cells, and MCF-10A breast epithelial cells. The results showed that the expression of *MTORT1* was lower in the breast cancer cells than in MCF-10A cells (**Figure 1C**), implying that *MTORT1* might function as a tumor suppressor. Next, to validate that *MTORT1* is a hypoxia-inducible lncRNA, quantitative RT-PCR was performed, resulting in similar changes in expression during hypoxia and re-oxygenation in MCF-7 and MDA-MB-231 cells (**Figure 1D**). In hypoxia mimic conditions, when cells were treated with CoCl_2_ under normoxia, the relative expression levels of *MTORT1* were also up-regulated in MCF-7 and MDA-MB-231 cells (**Figure 1E**). Furthermore, we overexpressed *HIF1A* mutant and *HIF2A* mutant, which are resistant to O_2_-regulated prolyl hydroxylation in the oxygen-dependent degradation domain, and therefore resistant to VHL-mediated ubiquitination and degradation, to examine their effects on *MTORT1* expression. *HIF1A* and *HIF2A* mRNA were successfully overexpressed under normoxia, and the relative expression levels of *MTORT1* were also up-regulated in MCF-7 cells overexpressing the HIF-1α P402A/P564A mutant (**Figure 1F**) or the HIF-2α P405A/P531A mutant (**Figure 1G**). These results demonstrated that the mitochondrial lncRNA *MTORT1* is an oxygen-responsive lncRNA that is up-regulated under hypoxia and down-regulated under re-oxygenation in breast cancer cells.

**Figure 2:**
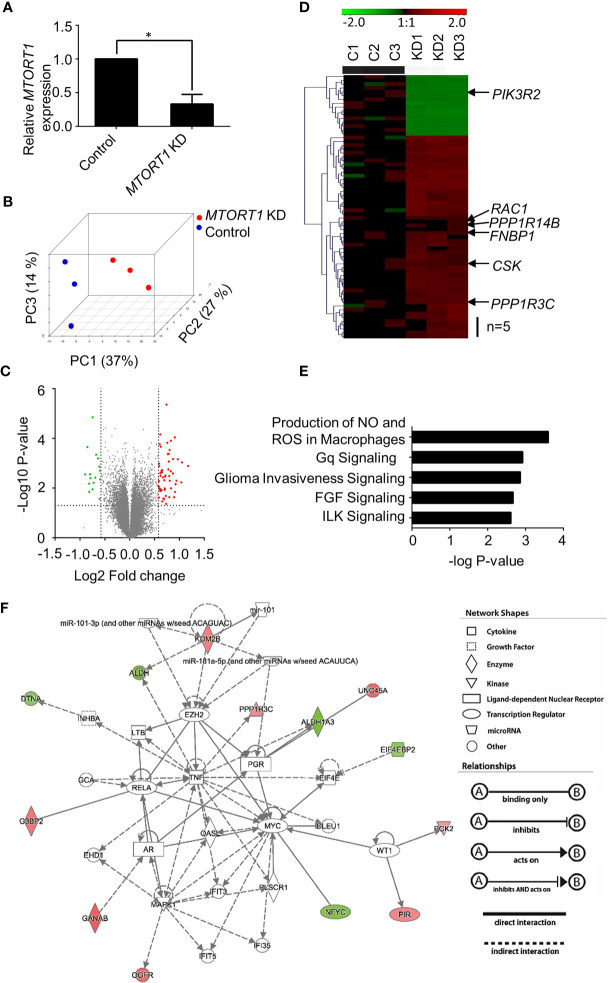
Identification of *MTORT1*-downstream genes by microarray analysis. **(A)** Relative expression levels of *MTORT1* in MCF-7 cells treated with siRNA against *MTORT1.* Expression levels were measured by quantitative RT-PCR and normalized to 18S rRNA. *P < 0.05. **(B)** Principal component (PC) analysis of *MTORT1* knockdown (KD) cells. PCs were plotted by the expression profiles of differentially expressed probes after quantile normalization. Each dot represents a sample. **(C)** Volcano plot of differentially expressed genes in *MTORT1* knockdown cells. Red points: up-regulated genes in *MTORT1* knockdown cells; green points: down-regulated genes; gray points: non-significant genes. **(D)** Heatmap and hierarchical cluster analysis of *MTORT1*-downstream genes. The labeled genes were validated by quantitative RT-PCR. Red color: up-regulated genes in *MTORT1* knockdown cells; green color: down-regulated genes; black color, non-significant genes. **(E)** The top five canonical pathways that *MTORT1*-downstream genes were enriched in, according to Ingenuity Pathway Analysis. **(F)** Representative network of *MTORT1*-downstream genes involved in cellular development, cellular growth, and proliferation. Red shapes represent genes that were up-regulated in *MTORT1* knockdown cells; green shapes represent down-regulated genes.

Since *MTORT1* was a novel lncRNA, in order to investigate its function, the genes affected downstream of *MTORT1* were identified using microarrays. Total RNA was extracted from MCF-7 cells treated with siRNA against *MTORT1* under normoxia. Differentially expressed genes were identified by Illumina Human HT−12 v4 Bead Chips. As shown in **Figure 2A**, *MTORT1* RNA levels were successfully knocked down in MCF-7 cells. To illustrate the differing expression profiles between *MTORT1*-knockdown and control cells, principal component analysis was performed after quantile normalization. As shown in **Figure 2B**, the distribution between *MTORT1*-knockdown samples (red spots) and the controls (blue spots) was separate, indicating different expression profiles.

To determine differential gene expression, *MTORT1*-knockdown and control cells were examined by Student’s t test, and the criteria for *MTORT1*-downstream genes was fold change > 1.5 (log_2_ Fold change > 0.58) and a significant difference (*P* < 0.05) (**Figure 2C**). Sixty-nine genes met the criteria. Among these, 53 genes were up-regulated and 16 genes were down-regulated in *MTORT1* knockdown cells (**Figure 2D**). The functions of these differentially expressed genes were analyzed by Ingenuity Pathway Analysis. As shown in **Figure 2E**, the top five pathways that *MTORT1* is possibly involved in are Production of Nitric Oxide and Reactive Oxygen Species in Macrophages, Gq Signaling, Glioma Invasiveness Signaling, FGF Signaling, and ILK Signaling. In addition, network analysis showed that some of the *MTORT1*-regulated genes are involved in cellular development, cellular growth, and proliferation (**Figure 2F**).

**Figure 3:**
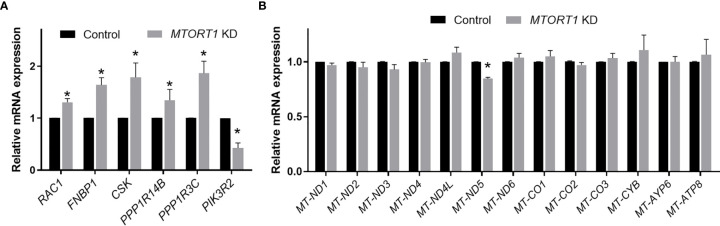
Expression profiles of *MTORT1*-downstream genes and mitochondrial genes in *MTORT1* knockdown cells. Relative expression levels of selected *MTORT1*-dwonstream genes **(A)** and mitochondrial genes **(B)** in MCF-7 cells treated with siRNA against *MTORT1* were measured by quantitative RT-PCR and normalized to 18S rRNA **(A)** or 16S rRNA **(B)**. The results are means ± SDs (n = 3). *P < 0.05.

To validate the results of the microarray analysis, the genes involved in the top 5 pathways and their interaction networks were measured by quantitative RT-PCR. The data of RT-PCR (**Figure 3A**) showed a similar pattern to those of microarray (**Figure 2D**) in *MTORT1* knockdown cells. Since microarray Illumina Human HT−12 v4 Bead Chips did not contain probes for mitochondrial genes, their expression levels in *MTORT1* knockdown cells were measured separately by quantitative RT-PCR. The results surprisingly revealed that only *MTND-5* was down-regulated in *MTORT1* knockdown cells (**Figure 3B**).

**Figure 4:**
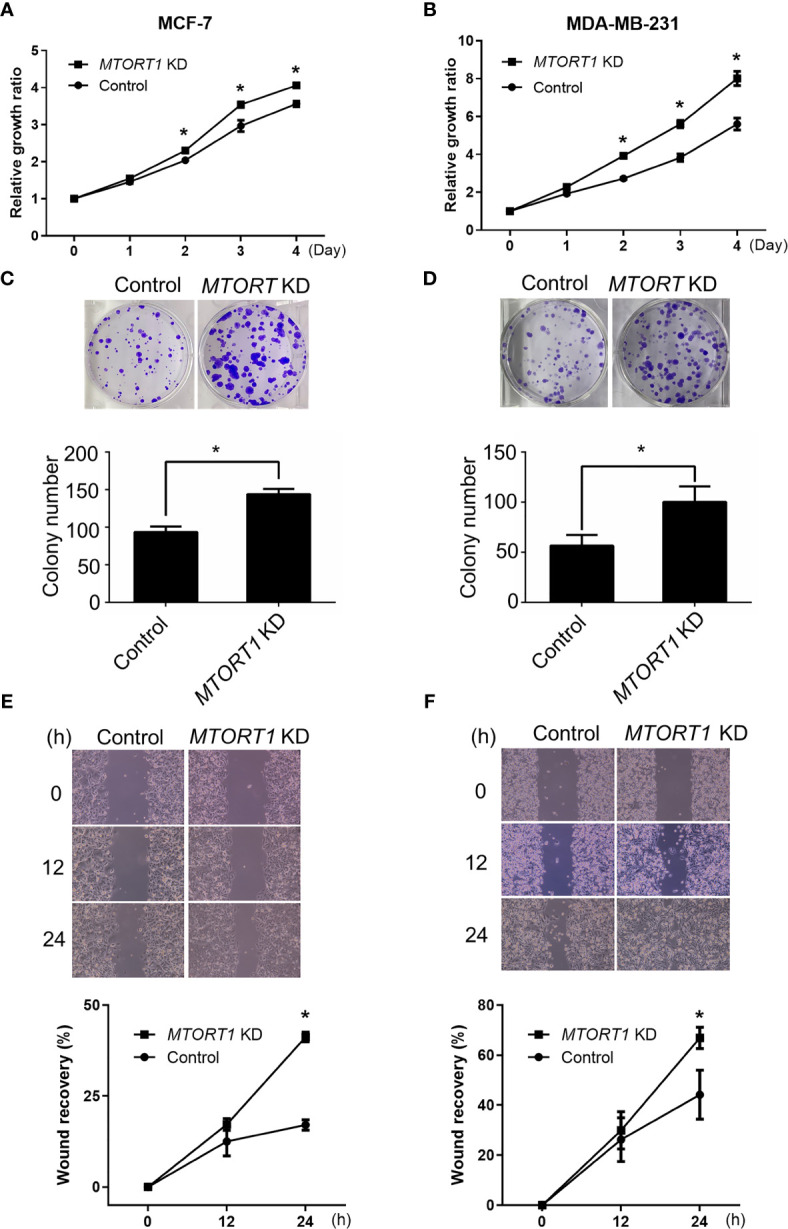
Knockdown of *MTORT1* enhances cell proliferation, migration, and invasion. **(A, B)** Cell proliferation using MTT assays. Cell growth was measured in MCF-7 **(A)** and MDA-MB-231 **(B)** cells treated with siRNA against *MTORT1*. The growth ratio was normalized to day zero. **(C, D)** Colony formation assay. Top: Representative images were taken of MCF-7 **(C)** and MDA-MB-231 **(D)** cells. Bottom: Quantification of colony counts. Colonies with cell numbers greater than fifty were counted. **(E, F)** Wound healing assay. Top: Representative images were taken of MCF-7 **(E)** and MDA-MB-231 **(F)** cells. Bottom: Quantification of wound recovery. The gap area was imaged at 0, 12, and 24 h. The percentage of wound recovery was compared to the wound area at 0 h. The results are means ± SDs (n = 3). *P < 0.05.

Since the results of the network analysis indicated that *MTORT1*-downstream genes were involved in cellular development, cellular growth, and proliferation (**Figure 2F**), the effects of *MTORT1* on cell proliferation were determined by MTT assays. The data showed a significant increment of relative growth ratio in MCF-7 (**Figure 4A**) and MDA-MB-231 (**Figure 4B**) cells treated with siRNA against *MTORT1*. In addition, knockdown of *MTORT1* facilitated colony formation in MCF-7 (**Figure 4C**) and MDA-MB-231 (**Figure 4D**) cells. These results indicated that *MTORT1* has suppressive effects on cell proliferation and colony formation in breast cancer cells.

Next, the effects of *MTORT1* on cell mobility were examined by wound healing assays. The results revealed that knockdown of *MTORT1* significantly promoted the migration ability of MCF-7 (**Figure 4E**) and MDA-MB-231 (**Figure 4F**) cells. These results suggested that *MTORT1* inhibits cell migration.

**Figure 5:**
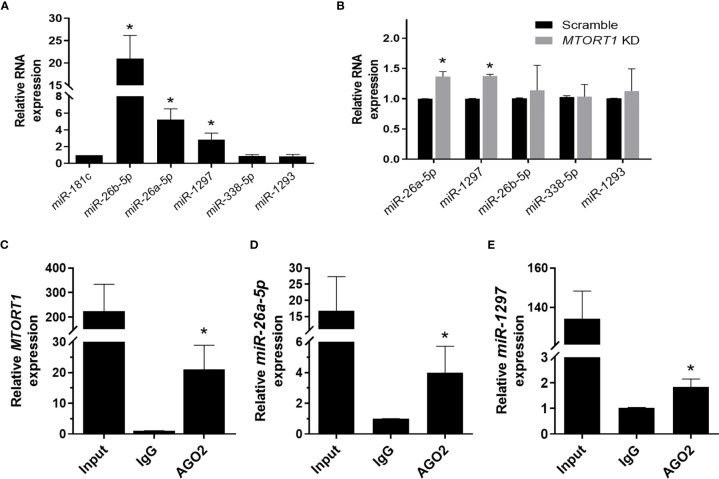
*MTORT1* serves as a miRNA sponge. **(A)** Enrichment of miRNA candidates in mitochondria in MCF-7 cells. Candidates of miRNA were predicted using miRDB (26). Relative expression levels of miRNA were measured by quantitative RT-PCR and compared to that of *miR-181c*, which was reported as a mitochondrial miRNA. **(B)** Relative expression levels of miRNA candidates in *MTORT1* knockdown cells were measured by quantitative RT-PCR and normalized to 18S rRNA. **(C–E)** RNA immunoprecipitation analysis of *MTORT1* **(C)**, *miR-26a-5p* **(D)**, and *miR-1297* **(E)** using antibody against AGO2 in MCF-7 cells. Relative expression levels of AGO2-enriched non-coding RNA were measured by quantitative RT-PCR and compared to those pulled down by IgG. The results are means ± SDs (n = 3). *P < 0.05.

Since RNA transcripts can function as miRNA sponges, we next explored whether the novel lncRNA *MTORT1* had such a function. First, we predicted the miRNAs that *MTORT1* possibly bound and their binding sites on *MTORT1* using miRDB (26). Among the predicted miRNA candidates, the relative abundance of the miRNAs expressed in mitochondria were measured by quantitative RT-PCR. Among them, *miR-181c* was reported to localize mainly to the mitochondria (27), and was chosen as a positive control. As shown in **Figure 5A**, *miR-26b-5p*, *miR-26a-5p*, and *miR-1297* were significantly (*P* < 0.05) enriched in the mitochondria of MCF-7 cells. Furthermore, the relative expression levels of *miR-26a-5p* and *miR-1297* were up-regulated in *MTORT1* knockdown cells (**Figure 5B**). These results indicated that *MTORT1* might serve as a miRNA sponge for *miR-26a-5p* and *miR-1297*. To further explore this, we examined the interaction between *MTORT1* and AGO2, an essential component of the RNA-induced silencing complex, which incorporates miRNA to interfere with RNA. RIP assays using anti-AGO2 antibody were performed, followed by quantitative RT-PCR. *MTORT1* (**Figure 5C**), *miR-26a-5p* (**Figure 5D**), and *miR-1297* (**Figure 5E**) were all significantly (*P* < 0.05) enriched in AGO2 immunoprecipitates as compared to those of IgG. Taken together, these results demonstrated that *MTORT1* could serve as a miRNA sponge.

**Figure 6:**
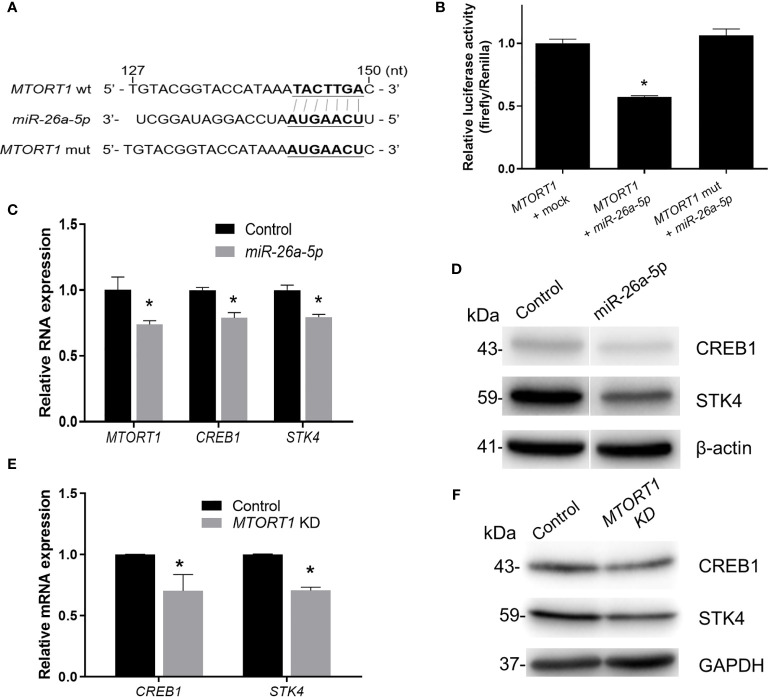
*MTORT1* up-regulates *CREB1* and *STK4* by directly interacting with *miR-26a-5p*. **(A)** Schematic diagram of the 3’ UTR of firefly luciferase constructs showing the sequence of *MTORT1* and the mutation of the *miR-26a-5p* binding site. The putative binding sequence was predicted by miRDB (26). wt, wild-type; mut, mutation. **(B)** Luciferase reporter assays in HEK-293T cells overexpressing *miR-26a-5p*. HEK293T cells were co-transfected with pMIR-REPORT-*MTORT1* or its mutant (pMIR-REPORT-*MTORT1* mutant), *miR-26a-5p* mimics, and *Renilla* luciferase plasmid (pGL4.74 [hRluc/TK]). Activity of firefly luciferase was normalized to the activity of *Renilla* luciferase. **(C)** Relative expression levels of *MTORT1* in HEK293T cells overexpressing *miR-26a-5p* were measured by quantitative RT-PCR and normalized to 18S rRNA. Relative expression levels of *miR-26a-5p* downstream genes *CREB1* and *STK4* are also shown. **(D)** Western blotting of CREB1 and STK4 in HEK293T cells overexpressing *miR-26a-5p*. β-actin: loading control. **(E)** Relative expression levels of *miR-26a-5p* downstream genes, *CREB1* and *STK4*, in MCF-7 cells treated with siRNA against *MTORT1*. The results are means ± SDs (n = 3). *P < 0.05. **(F)** Western blotting of CREB1 and STK4 in MCF-7 cells treated with siRNA against *MTORT1*. GAPDH: loading control.

Furthermore, to investigate the effects of the sponged miRNA on *MTORT1*, we chose *miR-26a-5p* for further experiments. First, *MTORT1* was inserted into the 3’ UTR of the luciferase gene in the pMIR-REPORT vector, and the predicted binding site of *miR-26a-5p* (143-149 nt) was changed to its complementary bases (pMIR-REPORT*-MTORT1* mutant) (**Figure 6A**). As shown in **Figure 6B**, overexpression of *miR-26a-5p* reduced the luciferase activity of pMIR-REPORT*-MTORT1*, whereas it failed to reduce the luciferase activity of the pMIR-REPORT*-MTORT1* mutant (**Figure 6B**). Furthermore, *MTORT1* was down-regulated in HEK293T cells overexpressing *miR-26a-5p* (**Figure 6C**). These data suggested the reciprocal inhibition of *MTORT1* and *miR-26a-5p*.

In addition, we predicted the target genes of *miR-26a-5p* using TargetScan 7.2 (28). Further examining the predicted target genes and the results of *MTORT1*-downstream genes using microarray analysis (**Figure 2D**), we found that *CREB1* and *STK4* might be the target genes of *miR-26a-5p*. The relative expression levels and protein amounts of *CREB1* and *STK4* were down-regulated in HEK293T cells overexpressing *miR-26a-5p* (**Figures 6C, D**). Also, the mRNA and protein amounts of *CREB1* and *STK4* were down-regulated in MCF-7 cells treated with siRNA against *MTORT1* (**Figures 6E, F**). Taken together, these results demonstrated that *MTORT1* serves as a miRNA sponge to regulate *CREB1* and *STK4* by directly interacting with *miR-26a-*5p.

## Discussion

In this study, we demonstrated that *MTORT1* is up-regulated under hypoxia and down-regulated under re-oxygenation in breast cancer. *MTORT1*-downstream genes were identified in MCF-7 cells treated with siRNA against *MTORT1* by microarray analysis. Sixty-nine genes were potentially regulated by *MTORT1*, and network analysis showed that *MTORT1* was possibly involved in cellular growth and proliferation. Functional assays revealed that knockdown of *MTORT1* enhanced cell proliferation and migration. Finally, RIP and luciferase reporter assays revealed that *MTORT1* serves as sponge of *miR-26a-5p* to up-regulate its target genes, *CREB1* and *STK4*.

In this study, we identified the oxygen-responsive mtlncRNA *MTORT1*, which was up-regulated under hypoxia (**Figures 1D, E**). Since *MTORT1* is a novel mtlncRNA, no study has reported it before. Although it was mainly located in mitochondria, the fraction of *MTORT1* in the nucleus under hypoxia (**Figure 1B**) was less than under normoxia (**Figure 1A**), implying that *MTORT1* may serve as a retrograde signal, facilitating crosstalk between the mitochondria and the nucleus, like SncmtRNAs and ASncmtRNAs (21). *MTORT1* was up-regulated in aerobic cells overexpressing *HIF1A* mutant and *HIF2A* mutant, suggesting that *MTORT1* is regulated by HIF-1α and HIF-2α (**Figures 1F, G**). These results showed that *MTORT1* may play a role under hypoxia. Therefore, to explore the function of *MTORT1*, we used microarrays to identify downstream genes of *MTORT1*. The top five pathways that *MTORT1*-downstream genes were involved in were production of nitric oxide and reactive oxygen species in macrophages, Gq signaling, glioma invasiveness signaling, FGF signaling, and ILK signaling (**Figure 2E**). The last four pathways are all involved in cancer progression. However, according to the network analysis, the differentially expressed genes involved in these pathways represent only about 5% of the total molecular content of each pathway. Examining the expression levels of mitochondrial mRNAs, only *MT-ND5*, encoding NADH dehydrogenase subunit 5 in a subunit of complex I, was down-regulated in *MTORT1*-knockdown cells (**Figure 3B**). These results suggested that *MTORT1* may not have much effect at the transcriptional level and that it may instead play a role at other levels, such as post-transcription, translation, or post-translation.

Among the *MTORT1*-regulated genes, the network analysis showed some of them were involved in cellular growth and proliferation (**Figure 2F**). Functional assays revealed that knockdown of *MTORT1* enhanced cell proliferation and migration (**Figure 4**), indicating that *MTORT1* acts as a tumor suppressor.

The existence of regulatory mechanisms between miRNAs and lncRNAs has been reported previously. LncRNAs can act as miRNA sponges to decrease the miRNA levels. Conversely, miRNAs can decrease the expression of lncRNAs through a mechanism similar to RNA interference (29). The cooperation between lncRNAs and miRNAs modulates gene expression *via* complex post-transcriptional mechanisms (30). Here, we also found that *MTORT1* could function as a miRNA sponge in mitochondria. Experimental validation showed that *miR-26a-5p* and *miR-1297* were enriched in mitochondria (**Figure 5A**), up-regulated in *MTORT1*-knockdown cells (**Figure 5B**), and bound with *MTORT1* in the RNA induced silencing complex (**Figures 5C, E**). Furthermore, luciferase reporter assays implied that *miR-26a-5p* could bind and inhibit *MTORT1* (**Figures 6B, C**). These results indicated that *MTORT1* served as a miRNA sponge and interacted with *miR-26a-5p*. However, how AGO2 and *miR-26a-5p* could selectively enter the mitochondria is still puzzling, and the mechanism of this relocation requires further investigation.

The expression levels of *miR-26a-5p*, *CREB1*, and *STK4* from The Cancer Genome Atlas (TCGA) dataset were examined by using ENCORI (starbase.sysu.edu.cn). Although negative correlation between *miR-26a-5p*-*CREB1* pair and *miR-26a-5p* - *STK4* pair was observed in some cancers, there was no significant negative correlation in aggressive breast cancer tissues (data not shown). Reasons of this discrepancy may be due to the differences between cell line and clinical tissues. Another reason may be due to the malignancy of breast cancer. A less aggressive breast cell line MCF-7 was examined in this study; however, the aggressive breast cancer tissues were examined in TCGA. Therefore, more experiments are needed to explore the regulatory mechanism of *CREB1* and *STK4* regulated by *miR-26a-5p*.

In this study, we identified a novel oxygen-responsive lncRNA, *MTORT1*, in breast cancer MCF-7 cells and characterized the functions of *MTORT1* and a regulatory mechanism of *MTORT1* in mitochondria. However, there were some limitations in this study. In order to investigate *MTORT1*-downstream genes, using both overexpression and silencing of *MTORT1* are optimal to reduce the false positives. However, when we tried to translocate *MTORT1* containing the *RP* sequence into mitochondria (31), the efficiency of translocation was very low. Furthermore, the effects of the *RP* sequence, which was reported to be transported into mitochondria, in cancer cells outweighed the functions of *MTORT1*. Hence, siRNA were used to knock down *MTORT1*.

## Conclusions

Our findings shed some light on the characterization, function, and regulatory mechanism of the novel hypoxia-induced mitochondrial lncRNA *MTORT1*. *MTORT1*-downstream genes are involved in cellular growth and proliferation of breast cancer cells. Lastly, *MTORT1* may serve as sponge of *miR-26a-5p* to up-regulate its target genes, *CREB1* and *STK4*. These data suggest that *MTORT1* may be a candidate for developing novel therapeutic regimen to inhibit breast cancer progression.

## Data Availability Statement 

The datasets presented in this study can be found in online repositories. The names of the repository/repositories and accession number(s) can be found below: https://www.ncbi.nlm.nih.gov/geo/, GSE157060.

## Ethics Statement

The study was approved by the Biosafety Committee of the College of Medicine, National Taiwan University [BG1050086].

## Author Contributions

Y-CC, L-LC, and L-CL conceived and designed the experiments. Y-CC and L-YS performed the experiments. Y-CC, L-LC, and T-PL analyzed the data. L-HC, EC, and M-HT contributed reagents, materials, and/or analysis tools. Y-CC and L-CL wrote the paper. All authors reviewed the manuscript. All authors contributed to the article and approved the submitted version.

## Funding

This work was supported by a grant from the Ministry of Science and Technology [MOST 109-2320-B-002-016-MY3, MOST-109-2314-B-182-030], and Chang Gung Memorial Hospital [CMRPD1H0611, CMRPD1I0141, CMRPD1I0142]. The funding source had no role in the design of this study and will not have any role in its execution or analysis, interpretation of the data, or the decision to publish the results.

## Conflict of Interest

The authors declare that the research was conducted in the absence of any commercial or financial relationships that could be construed as a potential conflict of interest.
